# Deadly Viruses With Beneficial Uses: The Poliovirus as Therapy

**Published:** 2013-09-01

**Authors:** Pamela Hallquist Viale

I have always been fascinated by books that focus on the plague. Worldwide infectious diseases have changed history multiple times. The original bubonic plague killed an estimated 25 million people in Europe in the late 1340s; in the Great Plague (1665-1666), London lost one in five residents to the disease. The Centers for Disease Control and Prevention (CDC) estimates that the plague carries a mortality rate of 66%, but with the advent of modern antibiotics, that rate has dropped to 8% to 10% (CDC, 2013a). The plague, caused by the bacterium Yersinia pestis, can progress to pneumonic plague, leading rapidly to death. It is unlikely that the most deadly form of this infection will occur again, as the pathogen has evolved to a lesser form. And although the death rate from the plague has been reduced dramatically since the introduction of antibiotics, the bacterium still exists, as its natural reservoir is the wild rodent. The plague probably will continue to be with us, with 1000 to 2000 cases each year, including some fatalities.

## Poliovirus

Poliovirus is an ancient virus that has caused global crippling and death. Although poliomyelitis was first recognized in 1789, outbreaks in Europe and the United States continued to occur with increasing severity (CDC, 2013b). This is a virus that could be eliminated permanently, as its reservoir is the human being (Dowdle & Birmingham, 1997). The peak of poliomyelitis infections occurred in 1952, with more than 21,000 paralytic cases. I have a personal interest in that outbreak: At that time, my sister-in-law became infected with a particularly virulent case of poliomyelitis, necessitating an iron lung for 6 months. She fought back and taught school from an electric wheelchair for 34 years. But the delayed effects and post-polio syndrome are now responsible for her decreased quality of life and significant pain and discomfort. With the possibility of global polio eradication occurring in the next decade, I couldn’t imagine keeping the virus around for any reason.

Yet a reason for keeping the poliovirus may indeed exist. One of the more intriguing reports from the 2013 American Society of Clinical Oncology (ASCO) annual meeting discussed a poliovirus vaccine trial for recurrent glioblastoma, a common and aggressive brain tumor. Preliminary data detailed the results of a phase I study involving seven patients who had recurrent glioblastoma (Dejardins et al., 2013). Three of those patients responded to the drug, which uses an engineered form of the virus to cause tumor cell death, avoiding normal cells. The virus-based therapy also stimulated the patients’ immune systems to attack the infected tumor cells as well.

Although the other patients in the study did not respond as well to the treatment, the three responders have had significant disease-free intervals (one is disease-free over 9 months after therapy, the second is 8 months out from therapy, and the third has had 2 months free of disease). Glioblastoma therapy is complicated by the inability to administer therapeutics beyond the blood-brain barrier; the experimental therapy described here was infused intratumorally by convection-enhanced delivery. This is an exciting report of a possible new therapy for a debilitating and often fatal tumor and a possible reason to keep a deadly virus alive.

## From Pathogen to Therapy

Novel antitumor treatments may not ever expose a patient with cancer to the bubonic plague, but there may be a reason to keep stockpiles of old and ancient enemies around. A recent report described using a version of the smallpox virus, vaccinia virus, in the treatment of triple-negative breast cancer in mice (Weaser, 2012). The treatment was effective in the infection and destruction of cancerous cells; angiogenesis of new tumor vessels was also blocked (Weaser, 2012). Although I worry about dangerous infectious viruses being used as a weapon in the wrong hands, the possibility of these previously devastating pathogens creating a beneficial therapy is intriguing, representing a reason to preserve even deadly diseases. 

## JADPRO LIVE!

You may have noted that JADPRO is holding its first educational symposium, JADPRO LIVE, on the 24th through 26th of January 2014 in sunny St. Petersburg, Florida! The editorial staff and our entire editorial board are extremely excited to be able to provide this conference. Our hope is that advanced practitioners will join us to discover new strategies for the care and treatment of patients with cancer as well as to have the opportunity to network with their peers. Topics will include important research and updates in the treatment of common cancers and symptom management.

We are especially pleased to announce that the keynote address will be given by Heather Hylton, physician assistant and former president of the Association of Physician Assistants in Oncology (APAO). Heather serves on ASCO’s Government Relations Committee and is an associate editor on the ASCO University Editorial Board. We are thrilled that the conference will start with a panel discussion on the relevance of the advanced practitioner in oncology care with perspectives shared by Dr. Peter Yu, the president-elect of ASCO for 2014-2015; Dr. Louis Harrison, former president of the American Society for Radiation Oncology; Robert Carlson, current CEO of the National Comprehensive Cancer Network and medical director of inpatient oncology and hematology at Stanford Cancer Institute; and Dr. Steven Allen, Chair of the American Society of Hematology’s Committee on Practice.

Please join us for what promises to be an exciting and unique forum for advanced practitioners to learn, discuss, and network on vital issues related to the care of our patients with cancer. I hope to see you there!
